# Autonomous oil flow generated by self-oscillating polymer gels

**DOI:** 10.1038/s41598-020-69804-3

**Published:** 2020-07-30

**Authors:** Kyosuke Yoshimura, Yuji Otsuka, Zebing Mao, Vito Cacucciolo, Takashi Okutaki, Hideto Yamagishi, Shinji Hashimura, Naoki Hosoya, Tasuku Sato, Yoko Yamanishi, Shingo Maeda

**Affiliations:** 1 Smart Materials Laboratory, Shibaura Institute of Technology, 3-7-5 Toyosu, Koto-ku, Tokyo, 135-8548 Japan; 2 Soft Transducers Laboratory, Institute of Microengineering, School of Engineering, École Polytechnique Fédérale de Lausanne (EPFL), Rue de la Maladière 71b, 2000 Neuchâtel, Switzerland; 3 Department of Engineering Science and Mechanics, Shibaura Institute of Technology, 3-7-5 Toyosu, Koto-ku, Tokyo, 135-8548 Japan; 4 Department of Mechanical Engineering, Kyusyu University, 744 Motooka, Nishi-ku, Fukuoka, 819-0395 Japan

**Keywords:** Chemical engineering, Polymer chemistry

## Abstract

The previously reported gel and polymer actuators require external inputs, such as batteries, circuits, electronic circuits, etc., compared with autonomous motions produced by the living organisms. To realize the spontaneous motions, here, we propose to integrate a power supply, actuators, and control into a single-component self-oscillating hydrogel. We demonstrate self-actuating gel pumps driven by the oscillatory Belousov–Zhabotinsky (BZ) reaction without electronic components. We have developed the volume oscillation of gels synchronized with the BZ reaction (BZ gel). Since the self-actuating gel pumps are driven by chemo-mechanical energy from BZ gels, the self-actuating gel pumps don’t require complex wiring designs, energy supply, and assembling. The mechanical work generated by BZ gels is extremely small. We formulated the thermodynamic cycle of BZ gels and maximized mechanical work. We found that pre-stretched BZ gel shows larger mechanical works. We physically separated the BZ gels and working fluid to create practical pumps. By using optimizing mechanical generated by BZ gels, we demonstrated the self-actuating gel pumps that transfer mechanical work through a stretchable elastomer membrane.

## Introduction

Machines and robots are essential to guarantee high production rates in industrial supply chains, while consumable electronics became ubiquitous in our daily lives. All these devices belong to the wide field of mechatronics, which encompasses different engineering technologies, such as mechanical, control, electronic, and information engineering. Advances in mechatronics in the last decades led to increasingly smaller and advanced robots and machines. However, such advancement came at the cost of a significant increase in the complexity and intricacy of these systems. It follows that engineers in industries can no longer develop and inspect machines without aids from a computer and other advanced diagnostic tools. Researchers and engineers developing robots and machines face increasing challenges in the design of systems composed by a large number of components, such as complex wiring designs, energy supply, and assembling. Aiming to overcome the complexity, we propose to use active and smart compliant materials that can solve complex tasks using few components and little computation^1,2^. Examples include self-stabilizing walkers that use the passive dynamics of their legs and soft grippers that adapt to the shape and stiffness of the grasped object.

**Figure 1:**
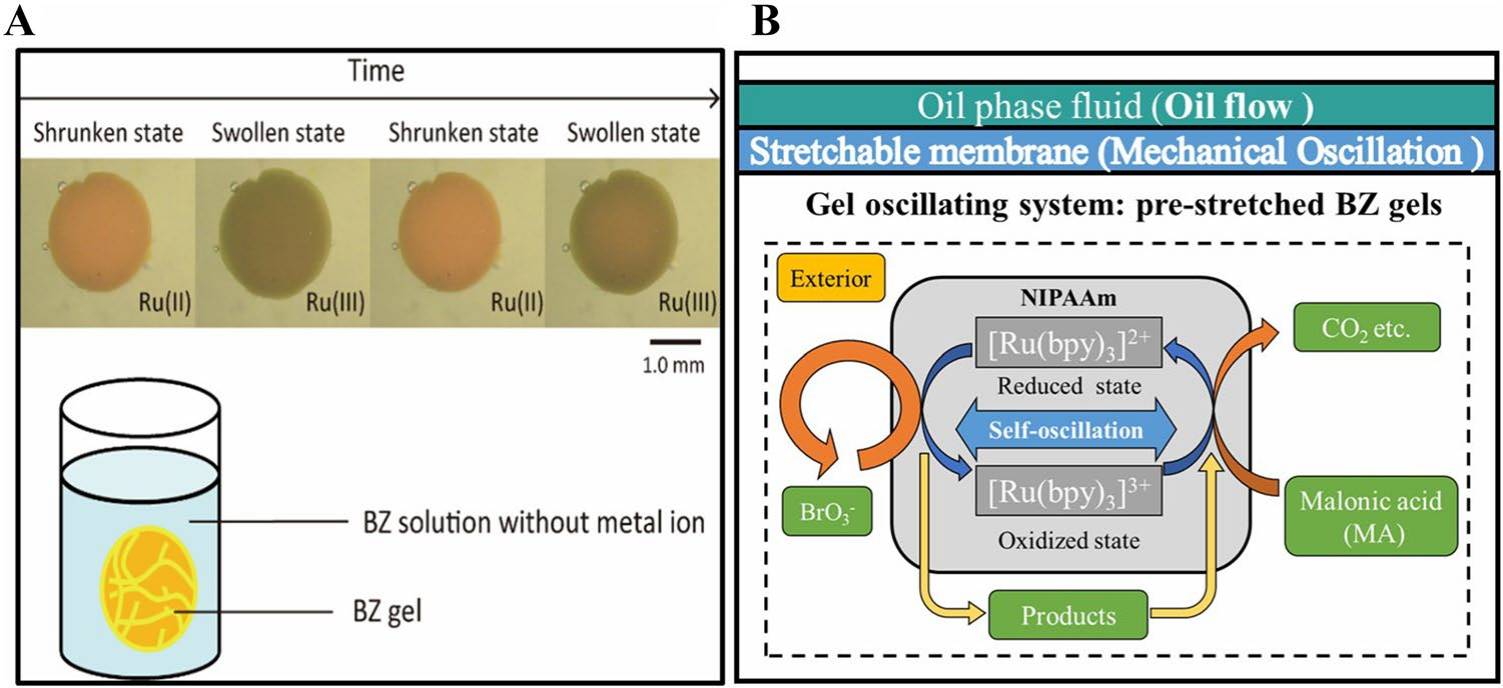
(**A**) BZ gel in the solution. (**B**) Conversion from BZ oscillation to oil flow through a membrane.

Herein we propose to move a step forward and blend the power supply, the actuators, and the control into a single-component self-oscillating hydrogel. Driven by chains of chemical reactions, these hydrogels oscillate by direct conversion of chemical energy into mechanical energy, alike self-motion in biological systems^3^. Hydrogels capable of self-motion have been presented in the previous work^4–8^. However, these oscillating hydrogels require being submerged in a strongly acidic chemical bath, which limits their practical applicability in mobile and wearable robotics. Here we present a method to extract mechanical work from encapsulated hydrogels (Fig. 1). A stretchable membrane successfully seals the gel with its chemical solution, while transferring the mechanical work to an external oil, used as a coupling medium. The results are self-actuating pumps that produce ready-to-use mechanical work from the autonomous chemical oscillations of hydrogels in the form of pressurized oil. These systems work without batteries, electronics, gears or bearings, leading to an extreme simplification compared to conventional electro-mechanical actuators. Similar to mammal’s hearts, these hydrogels push liquids back and forth using autonomous oscillations powered by chemical reactions. By driving the pressurized oil into soft fluidic actuators, such self-actuating pumps could be used as the core of mobile and wearable soft robots. The advantages would be the removal of any fluidic and electrical tethers and the ability to scale up the number of actuators without dramatically increasing the complexity of the system. Limitations regarding speed, force, and high-level control still need to be solved before these self-actuating pumps could be used in practical scenarios. However, the blending of all the components required by conventional electro-mechanical actuators (e.g., battery, electronics, microcontroller, electromechanical transducer, gears, bearings) into a single active material enables unprecedented simplification of the robotic system. Resembling the neuro-muscular apparatus of biological organisms, such self-actuating pumps bode to break the complexity wall faced by robotic systems with an increasing number of functions, enabling the development of truly smart multi-functional machines.

Most of the conventional chemical reactions proceed monotonically, with a reactant producing a product at a constant rate until the system finally reaches thermodynamic equilibrium. Differently, oscillating reactions, such as the Belousov–Zhabotinsky (BZ) reaction^9^, are non-monotonic. Such systems demonstrate oscillations and spatiotemporal variations in their reactant concentration. We demonstrated that a gel containing a BZ reaction (called BZ gel) that undergoes autonomous swelling–deswelling oscillations and peristaltic motion at the macroscopic scale. BZ gels harness a mechanism that couples BZ reaction and hydrogels to achieve autonomous motion. During the oxidizing reaction of the organic acid, the metal ions show redox-oscillation. In the BZ reaction, the concentration of some chemicals oscillates under stirred condition and chemical waves, as a reaction–diffusion system, which propagates under stationary conditions. Although the BZ reaction is called an oscillating reaction, the surface of the BZ solution remains always at the same level, while only the reaction propagates periodically. As a result, the BZ reaction generates spatio-temporal patterns.

Some researchers reported that polymers and gels undergo periodic chemo-mechanical oscillation when the BZ reaction occurs inside them^10^. Previously reported BZ gels capable of autonomous motion showed very small displacement, limiting their potential applications. Also, to the best of our knowledge, all the reported BZ gels have been tested only submerged in chemical baths^11–16^. In this work, we move a step forward in the practical use of BZ gels in robotic systems by demonstrating encapsulated BZ gels that transfer mechanical work through a stretchable elastomer membrane. Considering the BZ gels with their chemical solution as a thermodynamic system, the stretchable membrane plays the role of a movable boundary between the system and the external environment.

We develop a thermodynamic cycle of BZ gels at redox states. We then pre-stretch the BZ gels to improve the mechanical work obtained at each cycle. The whole gel with its chemical solution is encapsulated and the mechanical work produced by the swelling-contraction of the gel at each cycle is transferred to an external oil through the deformation of the elastomer membrane. The result is self-actuating pumps capable of moving a working fluid (environment) back and forth, following the oscillations of the BZ gel. We measured the motion of the fluid in a microchannel to quantify the performance of these pumps.

## Materials and methods

### Synthesis of samples

We prepared BZ gels by incorporating ruthenium monomer [Ru(bpy)_2_(4-vinyl-4′-methylbpy)]^2+^ ([Ru]^2+^; bpy = 2,2′-bipyridine) as a metal complex of the BZ reaction with stimuli-sensitive gel. The solubility of BZ gels in the oxidized [Ru]^3+^ state is slightly larger than that in reduced [Ru]^2+^ state, which leads to the volume change of BZ gels. Thus, BZ gels expand and contract periodically resulting from the redox oscillation of the metal complexion ([Ru]^2+^ ↔ [Ru]^3+^). We explored BZ gels with a significant difference in elastic modulus between the reduced and oxidized states of ruthenium ions using the micro-phase separation of NIPAAm polymer. We have synthesized ionic gels by copolymerization of NIPAAm and ruthenium monomers. First, we added 6.2 mg of *N*,*N*′-methylenebisacrylamide as a cross-linker, 71.2 mg of ruthenium(4-vinyl-4′-methyl-2,2′-bipyridine)bis(2,2′-bipyridine)bis(hexafluorophosphate) [Ru(bpy)_3_]^2+^, 573 mg of NIPAAm, and 8.7 mg of 2,2′-azobis(isobutyronitrile) as an initiator to 1.5 ml of methanol that was purged with nitrogen gas in advance. We mixed the solution and then stirred it for 15 min. Second, we added 18.2 mg of 2-acrylamide-2-methyl propane sulfonic acid to 1.5 ml of pure water that was purged with nitrogen gas in advance and then stirred the solution. We mixed the two solutions and stirred the mixture for 15 min. We injected the mixture into glass capillaries with an inner diameter of 1.0 mm. We put the glass capillaries into an oven at 60 °C for 12 h to obtain the polymerization of the solution. Once the polymerization process occurred, we submerged the synthesized gels in a methanol solution for one day to remove unreacted chemicals. Finally, we washed the gels in a series of the solution of methanol and pure water (concentrations of 100:0, 75:25, 50:50, 25:75, and 0:100%) for one day each.

We prepared the jigs made of poly(AAm-*co*-AAc) gels by photopolymerization at both ends of the BZ gels for connection with the testing apparatus. We bonded BZ gels and poly(AAm-*co*-AAc) gels together by creating an interpenetrating polymer network at the interface, as detailed in Fig. S1.

## Results and discussion

### Thermodynamic cycle of poly(NIPAAm-*co*-[Ru]-*co*-AMPS) gels

**Figure 2:**
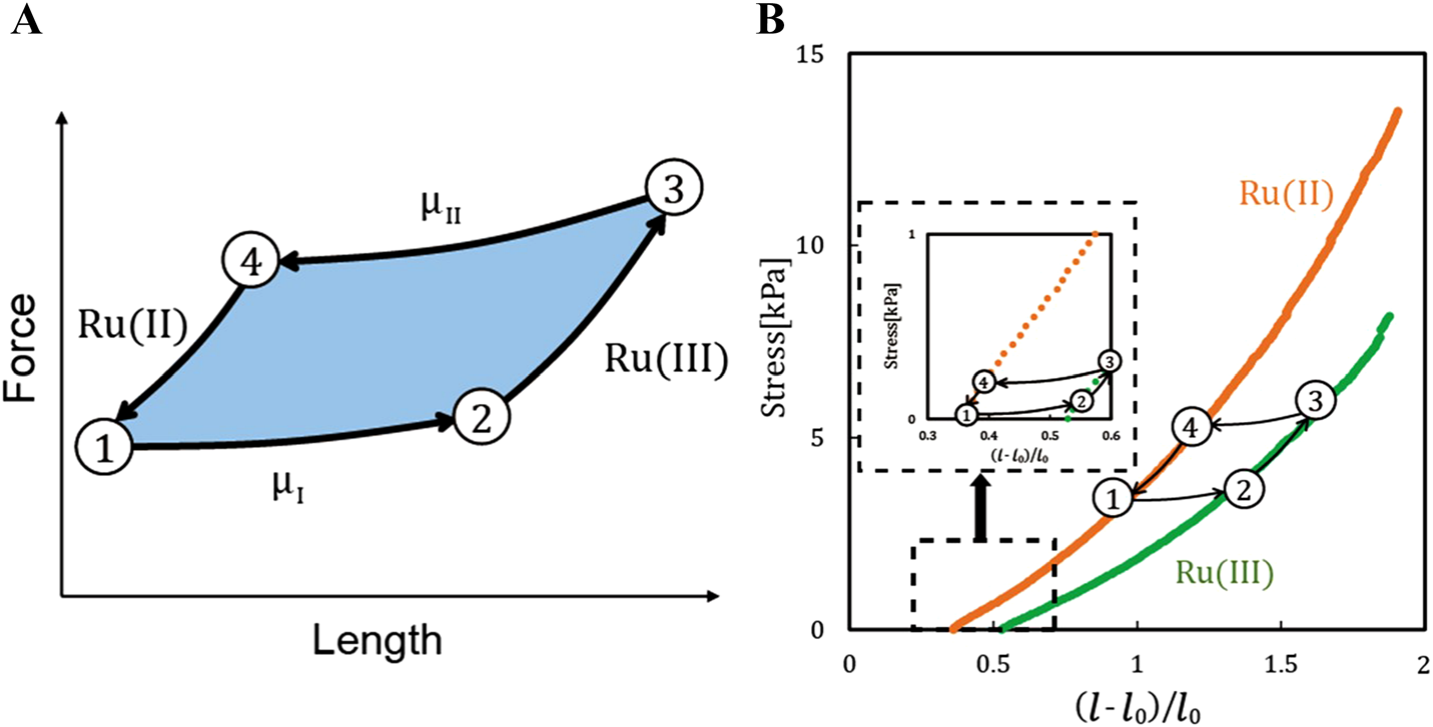
Thermodynamic cycle of poly(NIPAAm-*co*-[Ru]-*co*-AMPS) gel. (**A**) Force–length dependence of poly(NIPAAm-*co*-[Ru]-*co*-AMPS) gels at redox states. (**B**) Stress-extension ratio curves of BZ gels at 20 °C. Orange color indicates poly(NIPAAm-*co*-[Ru]-*co*-AMPS) gel at a reduced state. Green color indicates poly(NIPAAm-*co*-[Ru]-*co*-AMPS) gel at oxidized state.

To discuss the fundamental physical mechanism, we formulate the thermodynamic relation in redox poly(NIPAAm-*co*-[Ru]-*co*-AMPS) gels. Let us begin with reversible changes in length $dl$ generated by a redox reaction ([Ru]^2+^ ↔ [Ru]^3+^) in poly(NIPAAm-*co*-[Ru]-*co*-AMPS) gels. The work $S$ performed in the redox process is ${-}fdl$. Thermodynamic relation under isothermal and isobaric condition is described as follows (SI Appendix):

1 $$ S = - \oint {fdl} = \oint {\mathop \sum \limits_{i} \mu_{i} dn_{i} } = \int {\mathop \sum \limits_{i} \mu^{II}_{i} dn_{i} } - \int {\mathop \sum \limits_{j} \mu^{I}_{j} dn_{j} } , $$

where $\mu_{i} ,$ $dn_{i}$ are the chemical potential of *i*th chemical and the change in the number of moles of the *i*th chemical, respectively. The term on the left-hand side denotes the maximum mechanical work that can be obtained in the redox reaction. Figure 2A shows schematics of the thermodynamic cycle of the poly(NIPAAm-*co*-[Ru]-*co*-AMPS) gel. To obtain the thermodynamic cycle, we first soak the gel into an acidic solution with NaBrO_3_ as an oxidizing agent, with a chemical potential $\mu^{I}_{i}$ from the reduced state of the gel and let the gel swell along path 1–2. The gel is then mechanically stretched (2–3) in its oxidized state. Similarly, we obtain path 3–4 and 4–1 containing chemical potential $\mu^{II}_{i}$. The area $S$ encircled by the cycle 1–4 represents the work generated by the redox process. Thus, we can maximize the work in the redox process from the stress–strain curve of the redox poly(NIPAAm-*co*-[Ru]-*co*-AMPS) gels. To understand the system from the state equation of gel, we would mention the system at SI Appendix.

### Stress-extension ratio curves of redox poly(NIPAAm-*co*-[Ru]-*co*-AMPS) gels

**Figure 3:**
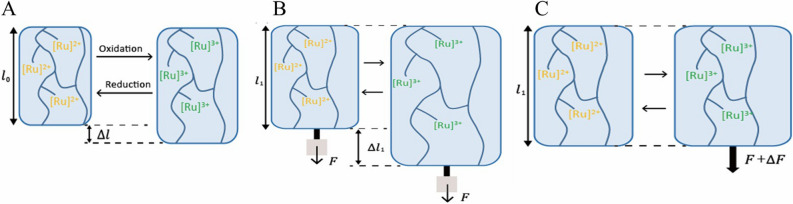
Equilibrium states of poly(NIPAAm-*co*-[Ru]-*co*-AMPS) gels under (**A**) free swelling, (**B**) applying a constant load, (**C**) constraining the length constant.

We developed a custom testing machine to measure the mechanical properties of the redox poly(NIPAAm-*co*-[Ru]-*co*-AMPS) gels (Fig. S2). Let us define $\sigma$ and $\varepsilon$ as the stress and the strain of BZ gels, respectively. The strain $\varepsilon$ is defined as $\left( {l - l_{0} } \right)/l_{0}$, where $l$ and $l_{0}$ are the length of poly(NIPAAm-*co*-[Ru]-*co*-AMPS) gels at equilibrium state and gelation state, respectively. Figure 2B displays the stress–strain curves of BZ gels in both reduced ([Ru]^2+^) and oxidized ([Ru]^3+^) states. The elastic modulus $\left( {\partial \sigma /\partial \varepsilon } \right)_{T}$ of the poly(NIPAAm-*co*-[Ru]-*co*-AMPS) gels in the oxidized ([Ru]^3+^) state is lower than in the reduced state. Also, as the strain of the gel increased, the stress difference between the reduced and oxidized states increased as well. As a consequence, pre-stretching the BZ gels leads to a larger work produced by the system at each cycle. It follows that also the force produced by a BZ gel would increase by using pre-stretch. The maximum work generated by a no pre-stretched BZ gel is represented by the area encircled by the ideal path 1–2–3–4 shown in Fig. 2B. We assessed the temperature dependency of the cycle and optimized the temperature (Fig. S3). In our system, the thermodynamic cycle is automatically driven by the oscillations of the BZ reaction. Figure 3 shows a schematic of the relation between the applied boundary conditions and swelling behavior of the BZ gels at thermodynamic equilibrium. BZ gels in free swelling generate limited displacement (Fig. 3A). Applying a constant load to the BZ gel leads to larger displacement (Fig. 3B) while constraining the length to a constant value results in large force-generation (Fig. 3C). We can use these findings to optimize the work that can be extracted from BZ gels.

### Measurements of force-generation of BZ gels

**Figure 4:**
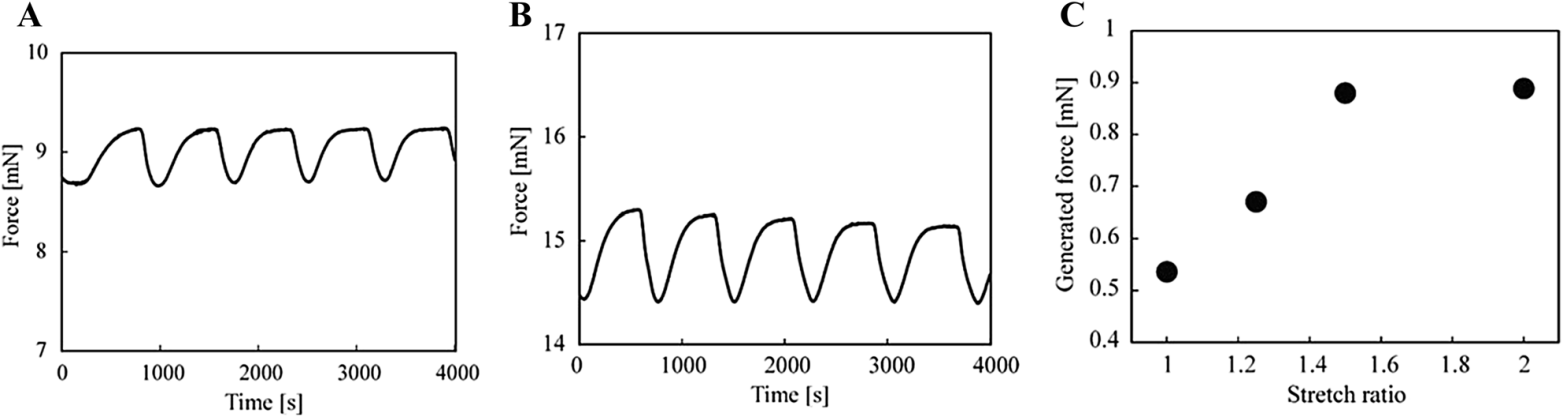
Force-generation of BZ gels. (**A**) Time profiles of no pre-stretched BZ gel. (**B**) pre-stretched BZ gel. (**C**) Force versus strain plots for BZ gels. Outer solution: [MA] = 0.06 M; [NaBrO_3_] = 0.06 M; [HNO_3_] = 1.2 M at 20 °C. The pre-stretched ratio is defined as ${\uplambda } = l/L$, where $l$ and $L$ are the stretched and initial length of the poly(NIPAAm-*co*-[Ru]-*co*-AMPS) gels at reduced [Ru]^2+^ state just before BZ reaction, respectively.

We investigated the relationship between the generated force and the length of BZ gels in redox states. The experimental setup is shown in Fig. S2. The apparatus consists of an automatic stage, a load cell, a thermo bath, and a manual stage. The load cell has a capacity of 50 mN (LVS-5GA, KYOWA Electronic Instruments Co., Ltd.). The BZ gel in our experiments has an initial diameter of 1.0 mm and an initial length of 12.0 mm. Figure 4 presents the oscillating profiles of the generated force of BZ gels. These results have been used in the design of the self-actuating pumps. We found that the generated force of BZ gels could be controlled through the pre-stretch ratio (Fig. 4A). As shown in Fig. 4B, the generated force of the BZ gel increases as the pre-stretched ratio $\lambda$ increases, until $\lambda$ = 1.5. The observed generated force of the pre-stretched BZ gel during the reaction was around 0.9 mN. The generated force of the pre-stretched BZ gel is 1.6 times higher than that of unstretched BZ gel. The generated pressure of the pre-stretched BZ gel is around 1.15 kPa. As shown in Fig. 4C, the generated force of the pre-stretched BZ gel has the saturation point at the following initial concentration.

**Figure 5:**
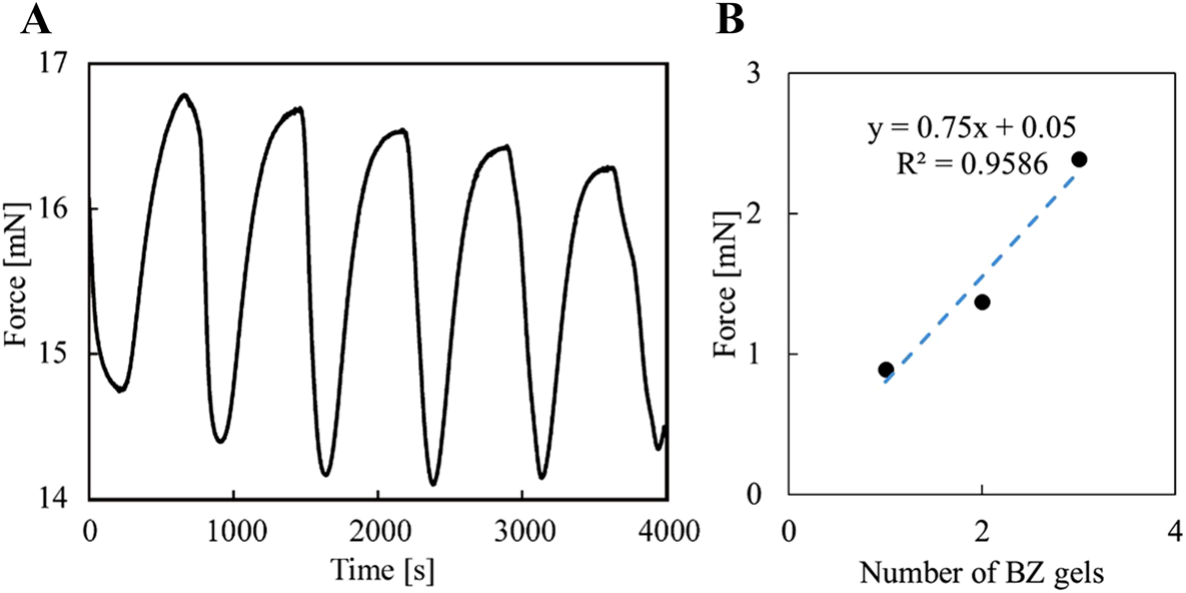
Force oscillation of multiple connected BZ gels in parallel. (**A**) Three BZ gels. (**B**) Force versus number of BZ gels. Outer solution: [MA] = 0.06 M; [NaBrO_3_] = 0.06 M; [HNO_3_] = 1.2 M at 20 °C.

We connected up to three pre-stretched BZ gels in parallel to obtain a greater force. Figure 5 shows an increasing force with the number of gels connected in parallel. We fitted the curve using the linear function, which is a line with a slope of 0.75 and y-intercept of 0.05 (Fig. 5B). The coefficient of determination is 0.9586. Three pre-stretched BZ gels produced around 2.4 mN. BZ reaction features several unique dynamics^17–21^, including a macroscopic synchronization between adjacent gels. We exploited such synchronization between our gels connected in parallel by leaving a small gap between them.

### Self-actuating pump

**Figure 6:**
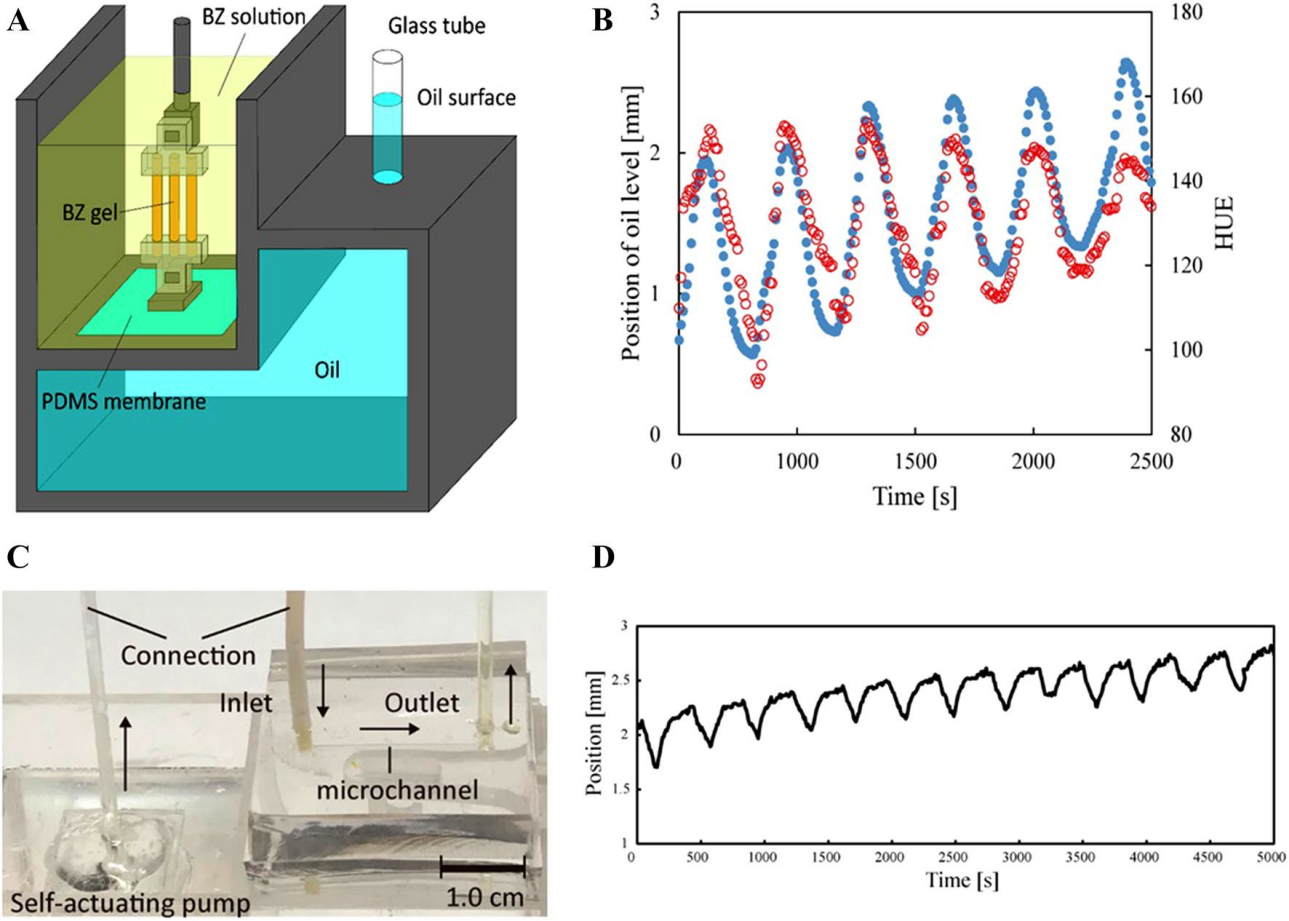
A self-actuating pump powered by BZ gel. (**A**) Schematic illustration of the pumps based on BZ gels. (**B**) Time profiles of the position of the oil level and average hue value of three BZ gels. (**C**) The self-actuating pump is connected with a microfluidic device. (D) Time profiles of the position of the oil level at the flow outlet. Outer solution: [MA] = 0.06 M; [NaBrO_3_] = 0.06 M; [HNO_3_] = 1.2 M at 20 °C.

Few efforts report the generation of the self-motion with chemical systems. Examples include self-motion of droplets^22^, self-motion of hydrogels^23^, and catalytic motors^24^ driven by chemical energy. All these self-moving systems share the common limitation of having being demonstrated only submerged in chemical baths. Recently, researchers have demonstrated the autonomous motion of soft robots based on the integration of fluidic logic gates and simple, monotonical chemical reactions^25^. While these systems produced autonomous motion driven by chemical reaction, they are still complex and comprising many components (e.g., reaction chamber, pinch-valves, exhaust valves). On the contrary, our self-actuating pumps rely on intrinsic chemical oscillations, removing the need for any valve and microfluidic circuit and opening to unconventional computation through chemo-mechanical networks. We experimentally demonstrated a self-actuating pump that is powered by BZ gels as shown in Fig. 6A. We physically separated the BZ gel system and a working oil using a thin stretchable PDMS membrane (thickness: 100 µm). We computed the expected fluidic flow of the working oil in our machine through FEM simulation (Fig. S4). The FEM simulations predicted a fluid flow of a few millimeters.

We tested the pumps by attaching one end of the pre-stretched BZ gels to the jig connected to the PDMS membrane. The BZ gels push and pull the PDMS membrane while it swells and shrinks, displacing the working oil back and forth. The actuator works under the condition with a constant pre-strained length (pre-stretched ratio λ = 1.5). During the oscillation process, the length changed with the redox reaction. The working mechanism of our pump is shown in Fig. S5. The observed pumping displacement during the BZ reaction was over 1.0 mm in a cylindrical tube (inner diameter: 1.0 mm). The average flow rate produced by our pump is 0.16 mm^3^/min. The experimental value of the fluidic flow is in good agreement with the FEM simulation (Fig. S4). We monitored the color change of three BZ gels and evaluated the hue value of it. The fluidic flow of the working oil synchronizes with the pre-stretched BZ gels as shown in Fig. 6B. Furthermore, we applied our self-actuating pump to the microfluidic device as shown in Fig. 6C. The width, height, and length in the microchannel are 800 µm, 70 µm, and 20 mm, respectively. We developed a microfluidic device including a straight micro-channel using PDMS (Polydimethylsiloxane). The micro-channel is connected to the self-actuating pump so that the oil can move back and forth through it following the periodic swelling and shrinking of the BZ reaction (Fig. 6C,D).

The oil position is primarily decided by the oscillation states of the BZ gels, membrane deflection, and damping of the oil fluid. In this case, the oil position keeps increasing is due to the membrane moves down further than that retracts back under the actuation of the three gels. In a period, the deflected membrane causes the oil to level up. The generated power of our actuator is estimated to 5.76 × 10^−2^ nW. Our future work will focus on the optimization of the system. There are two methods to optimize the system: physical and chemical methods. For the chemical method, the oscillated amplitude of the BZ gels increases with the growth of the period and amplitude of the redox changes. We can control the amplitude by changing the initial concentration of Malonic acid. For the physical method, we can change the beating rhythm of the gel by changing the solution temperature, changing the diameter of the gel, using different sequences to form the BZ reaction, the parallelized and seriated patterns of the gels, etc. Finally, we also consider optimizing the structures of the actuators.

## Conclusion

We have succeeded in obtaining useful mechanical work from BZ gels and self-actuating pumps without electrical circuits and programming. Beyond conventional mechatronics based on silicon and metal, soft and wet machines driven by chemical reaction networks blend a large number of electro-mechanical components into individual organ-like machines, showing great potential for simplification. Recently, toward a new direction of soft bio-inspired machines^26^, researchers developed electronic circuits made of organic semiconductors and synthetic resins to replace metals. We speculate that the true nature of the design is not merely in the choice of materials, but in the design, methodology to overcome the difficulties associated with the advancement of mechanical systems. Based on these findings, our self-actuating pump could operate as an artificial heart for soft machines.

## Supplementary information

Supplementary Information 1.

Supplementary Information 2.

Supplementary Information 3.

Supplementary Information 4.

## Data Availability

All data is available in the main text or the supplementary materials.
