# Supplementary material for: Autonomous oil flow generated by self-oscillating polymer gels
Source: Sci Rep. 2020 Jul 30;10:12834. doi: 10.1038/s41598-020-69804-3 (PMC7393118; doi:10.1038/s41598-020-69804-3)
Supplement: Supplementary file 1 — Supplementary Information 1. [file 41598_2020_69804_MOESM1_ESM.docx]

**SUPPLEMENTARY MATERIALS**

**Autonomous oil flow generated by self-oscillating polymer gels**

Kyosuke Yoshimura,^1^† Yuji Otsuka,^1,^ † Zebing Mao^1^*, Vito Caccuciolo,^2^

Takashi Okutaki,^1^ Hideto Yamagishi,^1^ Shinji Hashimura,^3^ Naoki Hosoya,^3^

Tasuku Sato,^4^ Yoko Yamanishi,^4^ Shingo Maeda,^1, 3^*

Correspondence to:
mao.zebing.v.5@sic.shibaura-it.ac.jp,
maeshin@shibaura-it.ac.jp

^1^Smart Materials Laboratory, Shibaura Institute of Technology, 3-7-5 Toyosu, Koto-ku, Tokyo 135-8548, Japan.

^2^Soft Transducers Laboratory, Institute of Microengineering, School of Engineering, École Polytechnique Fédérale de Lausanne (EPFL), Rue de la Maladière 71b, 2000 Neuchâtel, Switzerland.

^3^Department of Engineering Science and Mechanics, Shibaura Institute of Technology, 3-7-5 Toyosu, Koto-ku, Tokyo 135-8548, Japan.

^4^Department of Mechanical Engineering, Kyushu University, 744 Motooka, Nishi-ku, Fukuoka 819-0395, Japan.

Text

Fig. S1 Process for preparing BZ gel samples

Fig. S2 Testing machine for gels

Fig. S3 Stress-extension ratio curves of BZ gels

Fig. S4 FEM analysis

Fig. S5 Autonomous oil flow generator

Movie S1 Volume oscillation of a BZ gel (64x speed)

Movie S2 Three pre-stretched BZ gels in parallel (256x speed)

Movie S3 Oscillation of oil level driven by three pre-stretched BZ gels (256x

speed)


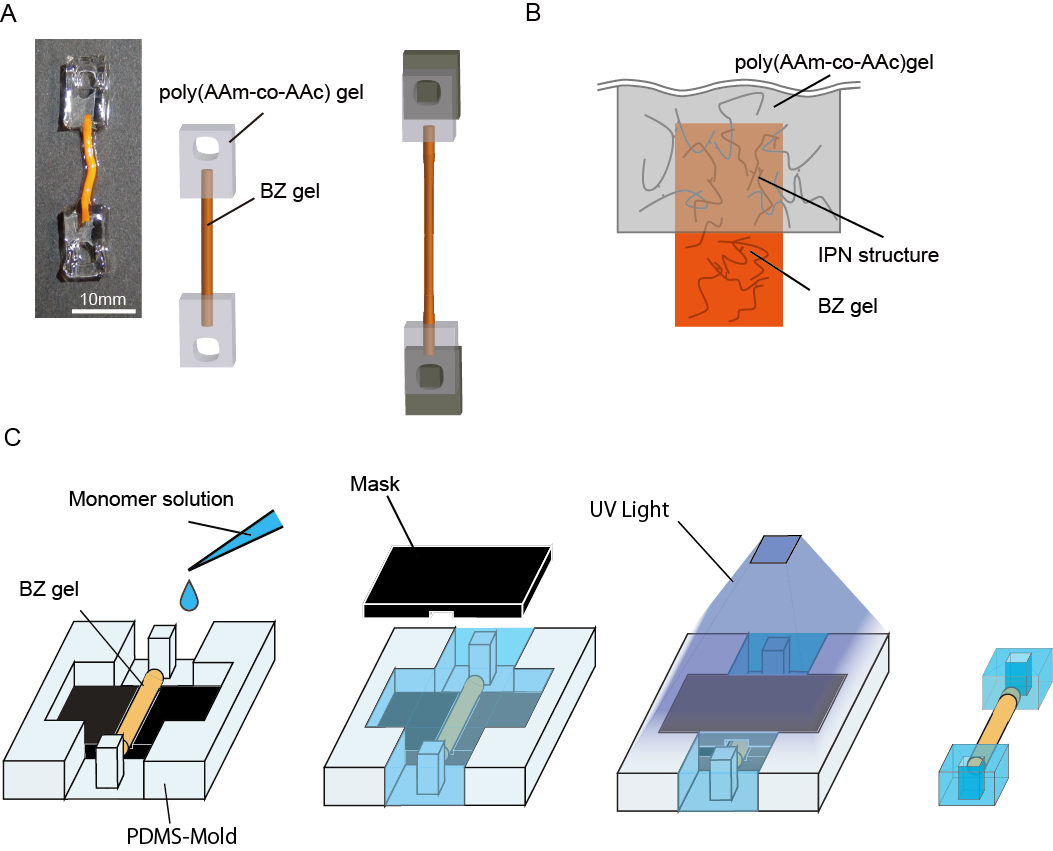


**Fig. S1** Process for preparing BZ gel samples. (A) BZ gel samples. (B) IPN structure of the sample. (C) Schematics of sample preparation.


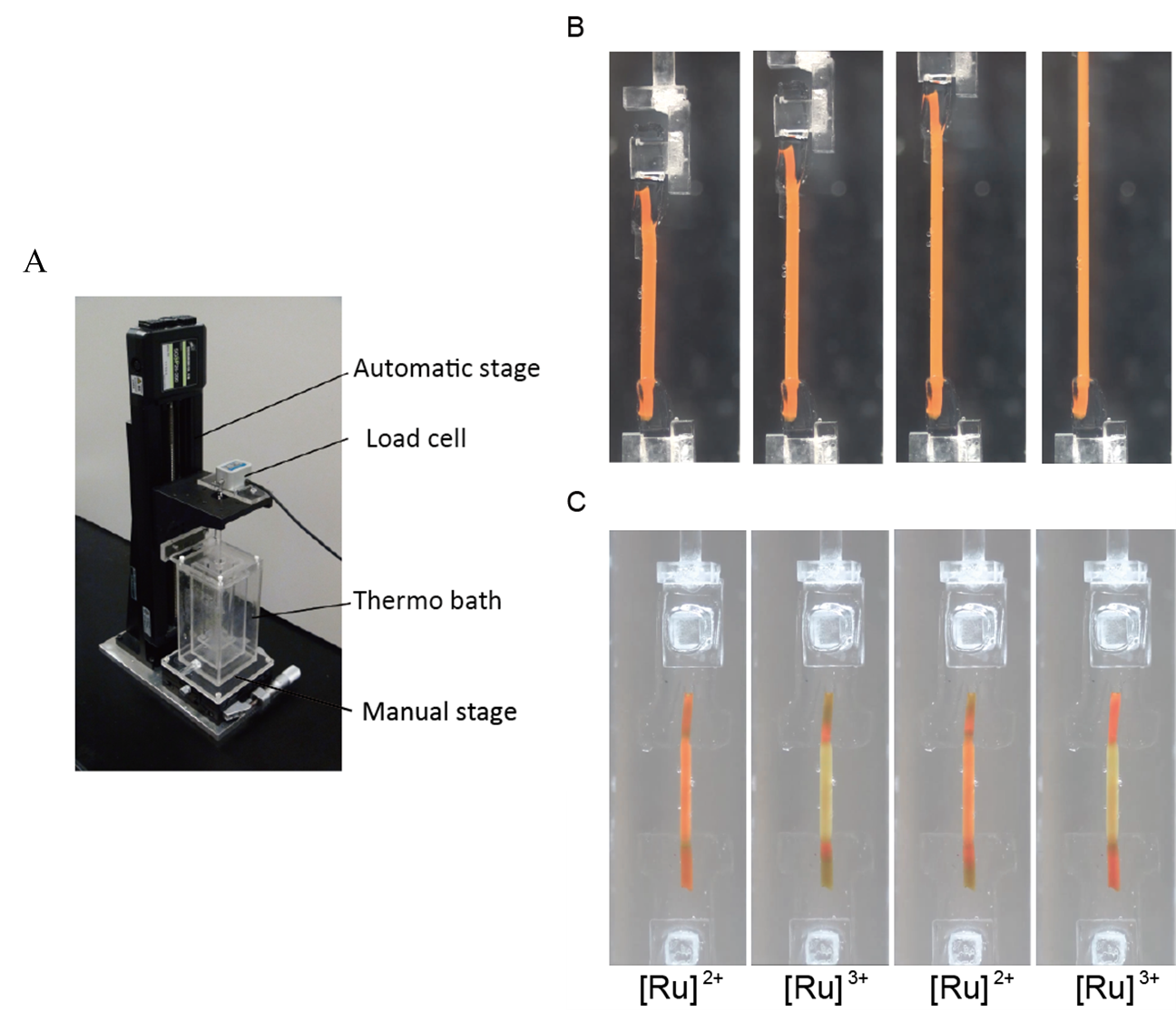


**Fig. S2** Testing machine for BZ gels. (A) Experimental setup. (B) Tensile test for poly (NIPAAm-co-[Ru]-co-AMPS) gel. (C) Stress change measurement of BZ gels under BZ reaction.

**Stress-extension ratio curves of BZ gels**

We investigated the temperature dependency of stress-strain curves of poly (NIPAAm -co-[Ru]-co-AMPS) gels. Figure S3 shows the stress-strain curves of poly (NIPAAm -co-[Ru]-co-AMPS) gels at 10ºC and 30 ºC, respectively. The elastic modulus of the oxidized poly (NIPAAm -co-[Ru]-co-AMPS) gel is a bit smaller than that of the reduced gel at 10ºC, and the gel at 10ºC has the same tendency as one at 20 ºC. Compared with the trend of poly (NIPAAm -co-[Ru]-co-AMPS) gel at 20 ºC, the stress difference between the gels at reduced and oxidized states at 20 ºC is larger than that at 10 ºC. The elastic modulus of the oxidized poly (NIPAAm -co-[Ru]-co-AMPS) gel is much larger than that of the reduced gel at 30ºC. Previously we reported that there are two states for mechanical oscillation of BZ gels, and they oscillate under either hydrophilic or hydrophobic states. The hydrophobic BZ gel generates very poor displacement because the redox potential of the metal ion in the BZ reaction is extremely small. The displacement of the BZ gels is proportional to the redox potential of the BZ reaction. Thus, we choose oscillating behavior at 20 ºC.


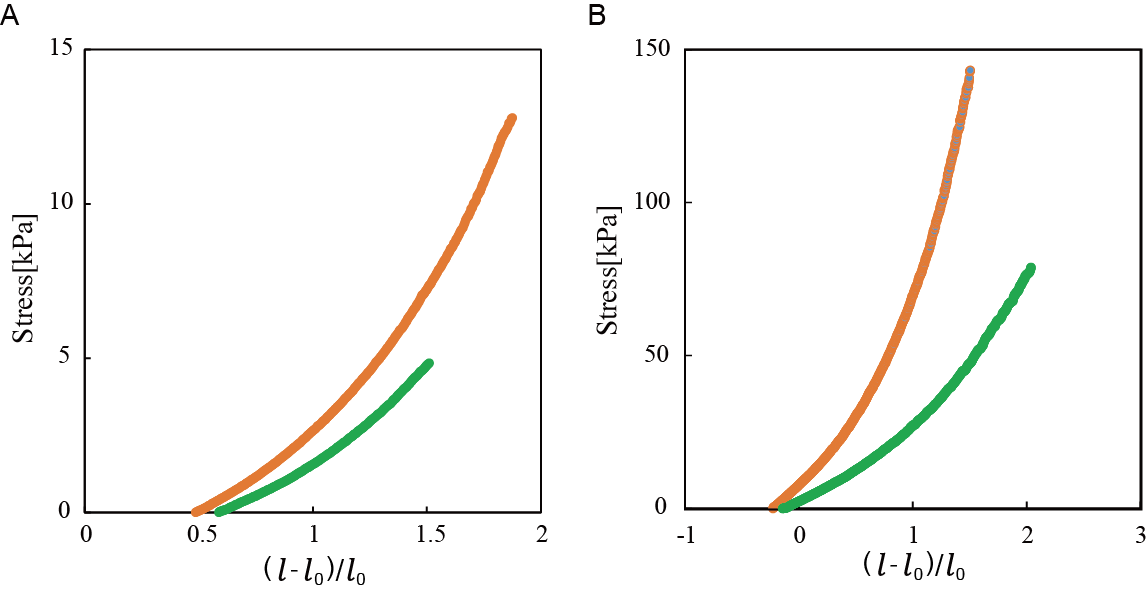


**Fig. S3** Stress-extension ratio curves of BZ gels at different temperature. (A) 10ºC. (B) 30 ºC.


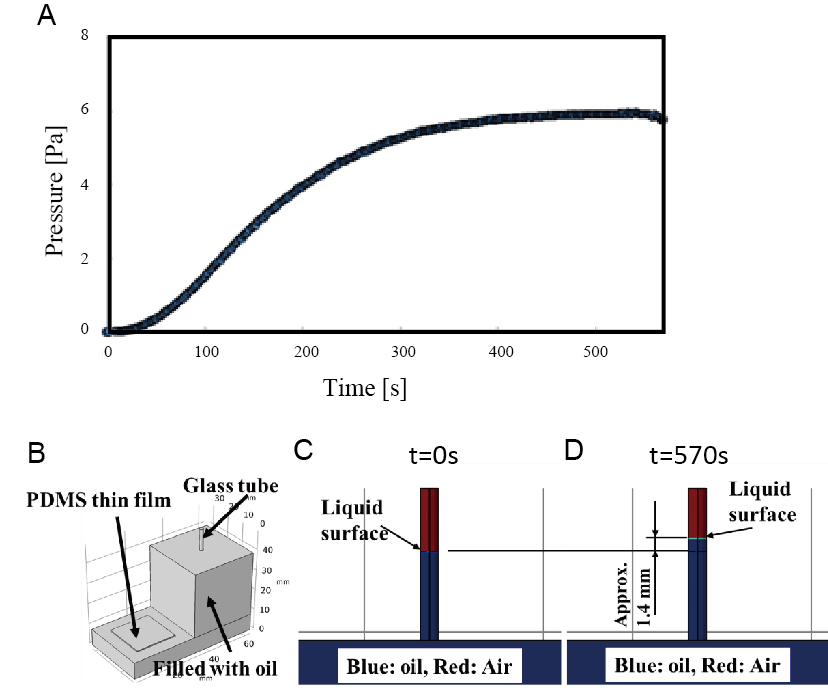


**Fig. S4** FEM analysis (A) Pressure function using the generated force of three BZ gels. (B) 3D model of BZ gels pump. (C) Applied pressure $P_{0}$= 375 Pa at t = 0 s. (D) Applied pressure $P_{0}$= 375 Pa at t = 570 s.


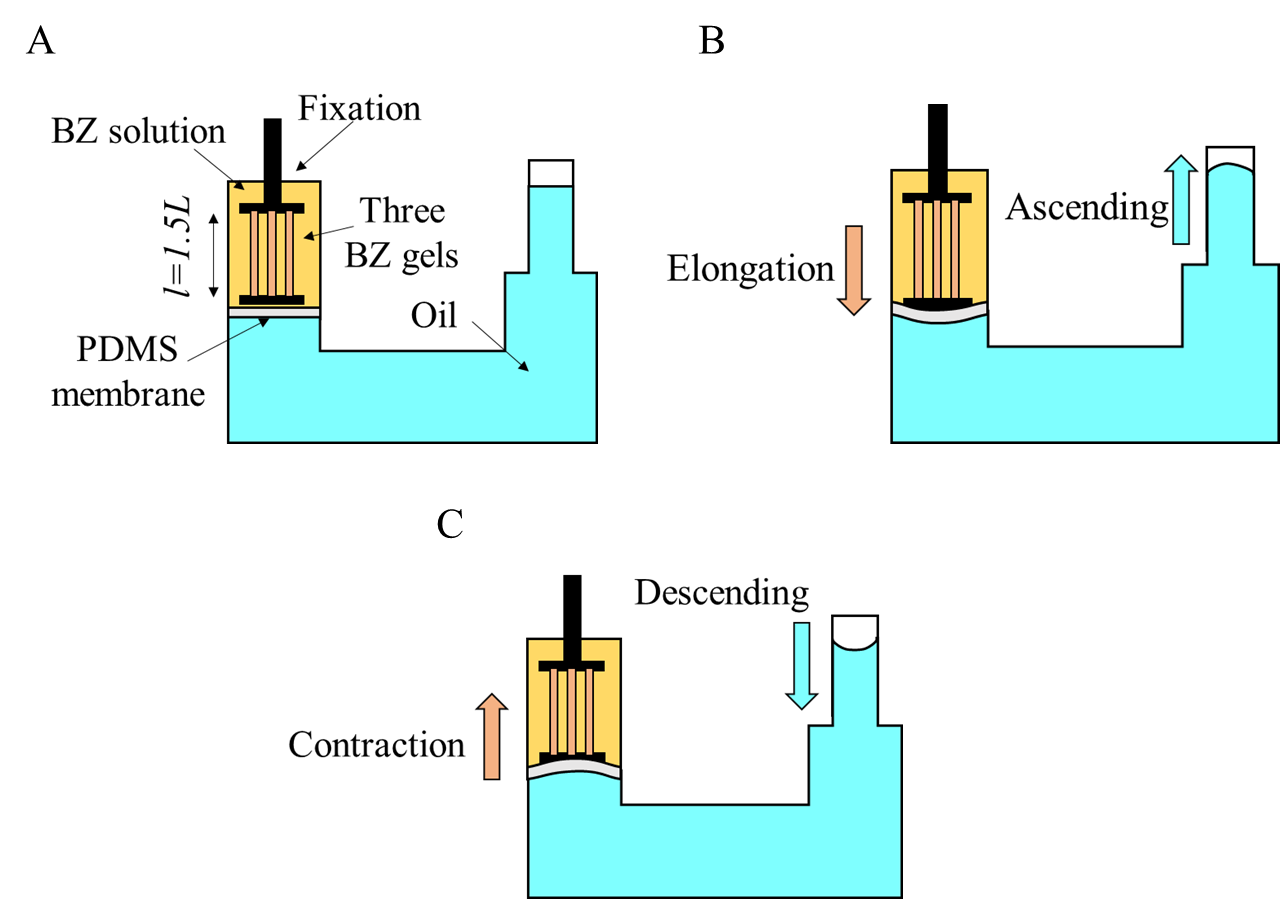


**Fig. S5** Autonomous oil flow generator. (A) Initial state of the gels system. (B) Oil level ascends when the gels elongate. (C) Oil level descends when the gels contract. *l* and *L* are the stretched and initial length of the poly (NIPAAm-co-[Ru]-co-AMPS) gels at reduced [Ru]^2+^ state just before BZ reaction, respectively.

**FEM simulation for BZ pump**

We found from the simulation that we can extract useful work from BZ gel by adjusting with hydrostatic pressure using the BZ solution. When the pressure applied to the system is small, the BZ solution is pushed by the pressure due to the oil's weight, and we can’t obtain sufficient liquid level displacement. When the pressure is large, the hydrostatic pressure of the BZ solution is sufficiently larger than the pressure generated by BZ gel. We used FEM analysis of the BZ gel pump using COMSOL to estimate the hydrostatic pressure that can obtain effective work. We modeled a BZ gel pump and performed a FEM analysis. We made pressure function $P_{gel}\left( t \right)$ using the force of BZ gels and the area of PDMS thin film obtained from our experiments, and gave them to a system as boundary stress. The equation to evaluate pressure $P\left( t \right)$ is expressed as follows.

$$P\left( t \right)=P_{gel}\left( t \right)+P_{0}=P_{gel}\left( t \right)+\rho gh_{w},$$

where *ρ*, *g*, and *h_w_* are the density of BZ solution, the gravity acceleration, and the distance between PDMS thin film and the solution level. The result shows that we can obtain the displacement of approximately 1.4 mm if $P_{0}=375 \mathrm{Pa}$ is applied. Assuming that BZ solution is 1M nitric acid, $\rho$ = 1063 kg/m^3^. If we evaluate the optimum height of BZ solution using relations between FEM analysis results and the equation $P_{0}=\rho gh_{w}$ shown in the above, $h_{w}$ is approximately 36 mm. Consequently, it indicates that three BZ gels efficiently generate useful work when the solution level is approximately 36 mm in height.

**The thermodynamic cycle of poly (NIPAAm -co-[Ru]-co-AMPS) gel**

The work performed in the redox process ([Ru]^2+^ ↔ [Ru] ^3+^) is expressed by the basic equation:

$$fdl=dU-TdS+PdV-\sum_{i} \mu_{i}dn_{i}.$$

In equation S1, $U$, $S,$are the inner energy and the entropy of the gel, respectively. We can rewrite the equation to understand the system of the redox process of the poly (NIPAAm-co-[Ru]-co-AMPS) gel in the following form:

$$\oint fdl=\oint dU-T\oint dS+P\oint dV-\oint\sum_{i} \mu_{i}dn_{i}.$$

The cyclic integral of a total differential must become zero. Thus, $dU$, $dS$, $dV$ are total differentials for a reversible process and their integrals over the thermodynamic cycle vanish. We then obtain the work performed in the redox process as follows:

$$S=-\oint fdl=\oint\sum_{i} \mu_{i}dn_{i}.$$
